# Social jetlag and diet quality among US young adults: interactions with race/ethnicity

**DOI:** 10.1017/jns.2024.18

**Published:** 2024-08-02

**Authors:** Xiru Lyu, Galit Levi Dunietz, Cindy W. Leung, Erica C. Jansen

**Affiliations:** 1 Sleep Disorders Center, Division of Sleep Medicine, Department of Neurology, Michigan Medicine, Ann Arbor, MI, USA; 2 Department of Nutritional Sciences, University of Michigan School of Public Health, Ann Arbor, MI, USA; 3 Department of Nutrition, Harvard T.H. Chan School of Public Health, Boston, MA, USA

**Keywords:** Circadian, Disparities, 24-hour recalls, Sleep, US, United States, NHANES, National Health and Nutrition Examination Study, MEC, Mobile Examination Center, HEI, Healthy Eating Index, NCI, National Cancer Institute, FPED, Food Patterns Equivalents Database, WTDR2D, dietary two-day sample weights

## Abstract

The objective was to examine associations between social jetlag and diet quality among young adults in the US using nationally representative data from the 2017–2018 NHANES survey, and evaluate effect modification by gender and race/ethnicity. Social jetlag was considered ≥2-hour difference in sleep midpoint (median of bedtime and wake time) between weekends and weekdays. Diet quality was assessed with the Healthy Eating Index (HEI)-2015 and its 13 dietary components. Ordinal logistic models were run with diet scores binned into tertiles as the outcome. Models accounted for potential confounders and survey weights. Effect modification by gender and race/ethnicity was examined. The study sample included 1,356 adults aged 20–39 years. 31% of young adults had social jetlag. Overall, there were no associations between social jetlag and diet quality. However, interaction analysis revealed several associations were race-specific (P, interaction<0.05). Among Black adults, social jetlag was associated with lower overall diet quality (OR = 0.4, 95% CI 0.2, 0.8; i.e. less likely to be in higher diet quality tertiles) and more unfavourable scores on Total Vegetables (OR = 0.6, 95% CI 0.3, 1.0) and Added Sugar (i.e. OR = 0.6, 95% CI 0.4, 0.9). For Hispanic adults, social jetlag was associated with worse scores for Sodium (OR = 0.6, 95% CI 0.4, 0.9) However, White adults with social jetlag had better scores of Greens and Beans (OR = 1.9, 95% CI 1.1, 3.2). Within a nationally representative sample of US young adults, social jetlag was related to certain indicators of lower diet quality among Black and Hispanic Americans.

## Introduction

In the US, poor diet is a contributing factor to most leading causes of death^(1)^ and morbidity, including cardiovascular disease,^(2)^ type 2 diabetes,^(3)^ cancer,^(4)^ and stroke.^(5)^ It is also related to weight gain^(6)^ and inflammation,^(7)^ risk factors for COVID-19 severity. Across all age groups, young adults are among the populations with the lowest quality diets in the US.^(8)^ This is alarming, since young adulthood diets tend to track over time and may be much harder to modify later in life.

There are multiple reasons for poor diet quality in young adulthood. First, young adulthood is a transitional life-stage with newfound independence and responsibility, which may include new jobs and careers, continuing education, marriage/partnerships, and children. These transitions and responsibilities could be associated with lower-quality diets for a number of reasons, including lack of time or knowledge of food preparation,^(9,10)^ lower access to healthy food due to financial insecurity,^(11,12)^ or higher preference for unhealthy foods due to stress and uncertainty.^(13,14)^

Sleep is emerging as an important predictor of dietary behaviour among young adults. Short sleep duration and poor quality sleep have each been related to lower diet quality among young adults.^(15)^ Consistency of sleep may also affect dietary habits,^(16)^ although it has not been examined as thoroughly. Moreover, the consistency of sleep timing from weekdays to weekends (‘social jetlag’), could be particularly relevant for young adults. It has been documented that young adults are more likely to have social jetlag,^(17)^ often defined as >2 hour difference in timing of weekend and weekday sleep.^(18)^ This association may be explained by the fact that adults in their early twenties typically have a circadian drive for eveningness^(19)^ and thus may be more likely to stay up late on weekends and shift to an earlier sleep weekday schedule to align with school or work. Moreover, social jetlag could be related to poor diet quality for several reasons. Late weekend bed and wake times could be directly tied to less-healthy diet patterns on weekends, including breakfast skipping, irregular meal-times, and late-night eating.^(20–22)^ In support, although sleep was not examined, nationally representative US data show that weekend eating patterns are less healthy overall than during the week.^(23,24)^ In addition, the constant shift in sleep schedules from weekends to weekdays could impact overall sleep quality and duration, which in turn relate to lower-quality diets. Indeed, studies among Spanish,^(25)^ Japanese,^(26)^ and Brazilian populations^(20)^ have reported associations between social jetlag and lower diet quality among young adults. Yet, to our knowledge no studies have been conducted with nationally representative data from the US. Further, it is important to consider potential interactions by sex and race/ethnicity, since there may be different contexts and norms regarding sleep and eating behaviours by these characteristics.^(27)^ Furthermore, there are known disparities in both diet quality and sleep health, with underrepresented populations often experiencing worse diet quality and sleep.^(28,29)^

Thus, in order to address the current gaps in the literature, we sought to examine the role of social jetlag in diet quality among young adults aged 20–39 years in the US. Because there is not a clear consensus on the ages that encompass young adulthood, we selected a wide range. We hypothesized that relationships with diet quality would differ by both sex and race/ethnicity.

## Participants and methods

### Study population: the national health and nutrition examination survey

First conducted during the early 1960s, the National Health and Nutrition Examination Survey (NHANES) aims to evaluate health and nutritional status of US children and adults. Since 1999, the NHANES programme has become a continuous annual survey that provides a nationally representative sample to study the US population of all ages. Each year, around 5000 randomly selected noninstitutionalized US residents participate in the survey, where they receive interviews and physical examinations. Deidentified data is then released to the public in 2-years cycles. The present analysis utilized 2017–2018 NHANES data to examine the relationship between social jetlag and diet behaviour among a restricted sample of adults of age 20–39 years (N = 1,308).

### Exposure: social jetlag status

In 2017–2018 NHANES, questions on sleep timing were adapted from the Munich ChronoType Questionnaire.^(30)^ Participants of age 16 and above were asked to report usual sleep and wake times on weekdays (or workdays) and on weekends (or non-workdays), respectively. These questions allowed us to assess individual social jetlag status, defined as the discrepancy in sleep timing between work and free days.^(18)^ Specifically, for each study participant, we calculated and compared weekday and weekend sleep midpoints (i.e. the median of sleep and wake times). Those with at least a 2-hour difference in sleep midpoints were categorized as having social jetlag. Since there is no standardized cut-off for defining social jetlag, we also considered a cut-off of 1 hour in post hoc analysis. However, we used the more stringent definition for our primary analysis since social jetlag is so prevalent in young adult populations.

### Outcome: healthy eating index

Two 24-hour dietary recall interviews were conducted for study participants to estimate intakes of energy and nutrients from consumed foods and beverages. The first interview was administered in person in the Mobile Examination Center (MEC), and the second one was over telephone 3–10 d later. We utilized the Healthy Eating Index (HEI-2015), a measure evaluating diet quality based on key recommendations of the *2015–2020 Dietary Guidelines for Americans,* to quantify participants’ diet behaviour.^(31)^ Rated with a scoring system, the HEI-2015 includes a total score that indicates the overall diet quality, and 13 component scores (i.e. total fruits, whole fruits, total vegetables, greens and beans, whole grains, dairy, total protein foods, seafood and plant proteins, fatty acids, refined grains, sodium, added sugars, and saturated fats) with each assessing one aspect of the *Dietary Guidelines*. All scores have a minimum of 0 points. The maximum is 100 points for the total score, which varies from 5 to 10 for component scores. Higher scores suggest better alignment between diet quality of food intakes and key recommendations by the *Dietary Guidelines*. Utilizing the ‘simple HEI scoring algorithm – per person’ method by National Cancer Institute (NCI),^(32)^ participant-specific HEI-2015 scores were calculated using the average of the first day and second day total nutrient intakes from the NHANES data and the Food Patterns Equivalents Database (FPED). Scores were then binned into three groups, using tertile values as cut-offs.

### Potential confounders

The following demographic and health characteristics were considered as potential confounders – age, gender (female/male), race/ethnicity (White, Black, Hispanic, Asian, other), educational attainment (below high school/high school degree and above), tobacco use in the last 5 d (yes/no), and typical engagement in work or recreational physical activity at least at the moderate level (yes/no).

### Statistical analysis

**Fig. 1.**
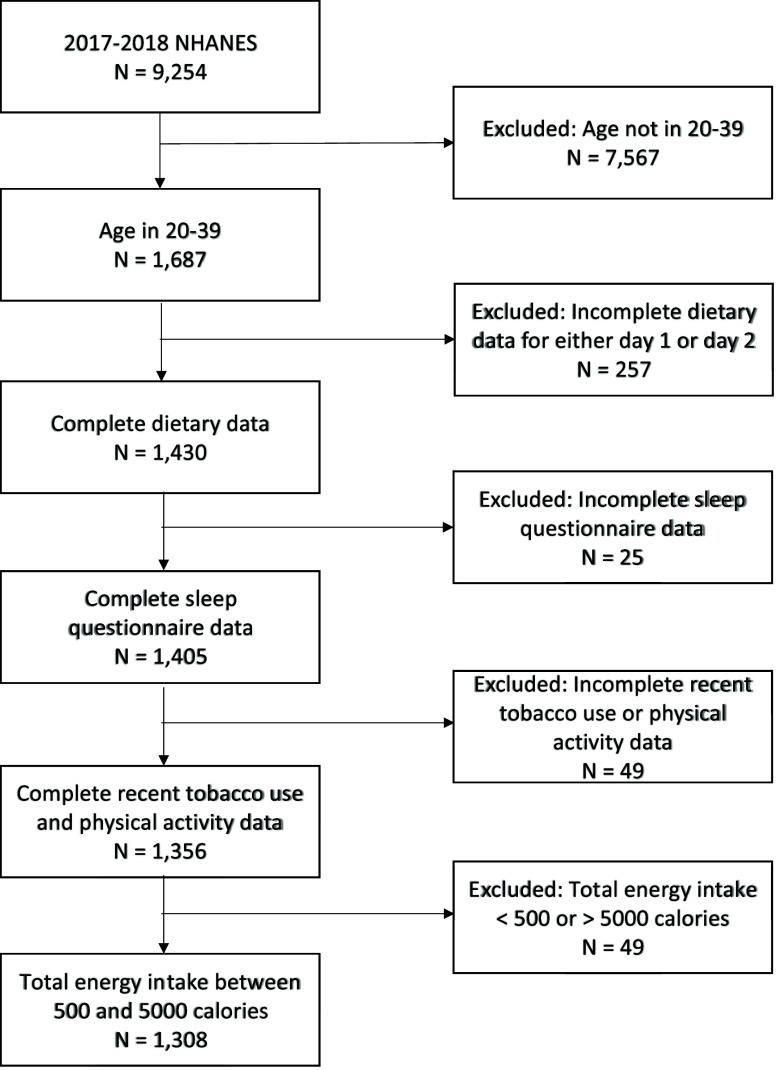
Title: Flowchart of the data included in the final analytic sample.

A total of 9,254 individuals participated in the 2017–2018 NHANES survey. Participants with incomplete dietary recall, sleep questionnaire, or potential confounders (recent tobacco use and physical activity were the only confounders with missing data) were excluded from the study. Furthermore, the analysis focused on young adults of age 20–39 years with total energy intake between 500 and 5000 energies (considered as plausible energy intake), resulting in an analytic sample of 1,308 participants (Fig. 1). Using complex survey analytic methods, demographic characteristics and personal habits were evaluated for all participants and by social jetlag status. Adjusted by the dietary two-day survey weight, bar charts were also generated to present gender-specific proportions of participants with social jetlag, for overall and race/ethnicity-stratified samples.

Associations between social jetlag and diet behaviour were examined with ordinal logistic regression models, with the tertiles of diet scores (overall and components) as categorical outcomes. Through incorporating the dietary two-day sample weight (WTDR2D) in the analysis, effect sizes and corresponding 95% CIs were estimated (R statistical software version 4.2.1, with the *survey* package); models included age, gender, race/ethnicity, educational attainment, recent tobacco use and physical activity as covariates. We considered BMI and sleep duration as potential mediators and therefore did not adjust for them.

We further performed interaction analyses by including an interaction term for social jetlag by gender and social jetlag by race/ethnicity. Because we saw evidence for effect modification (P, interaction<0.05) by race/ethnicity, we finally ran race/ethnicity-stratified analyses. Race/ethnicity-stratified analyses are shown only for Whites, Blacks, and Hispanics due to insufficient sample sizes in the other race/ethnicity categories (Asian and other).

## Results

**Fig. 2.**
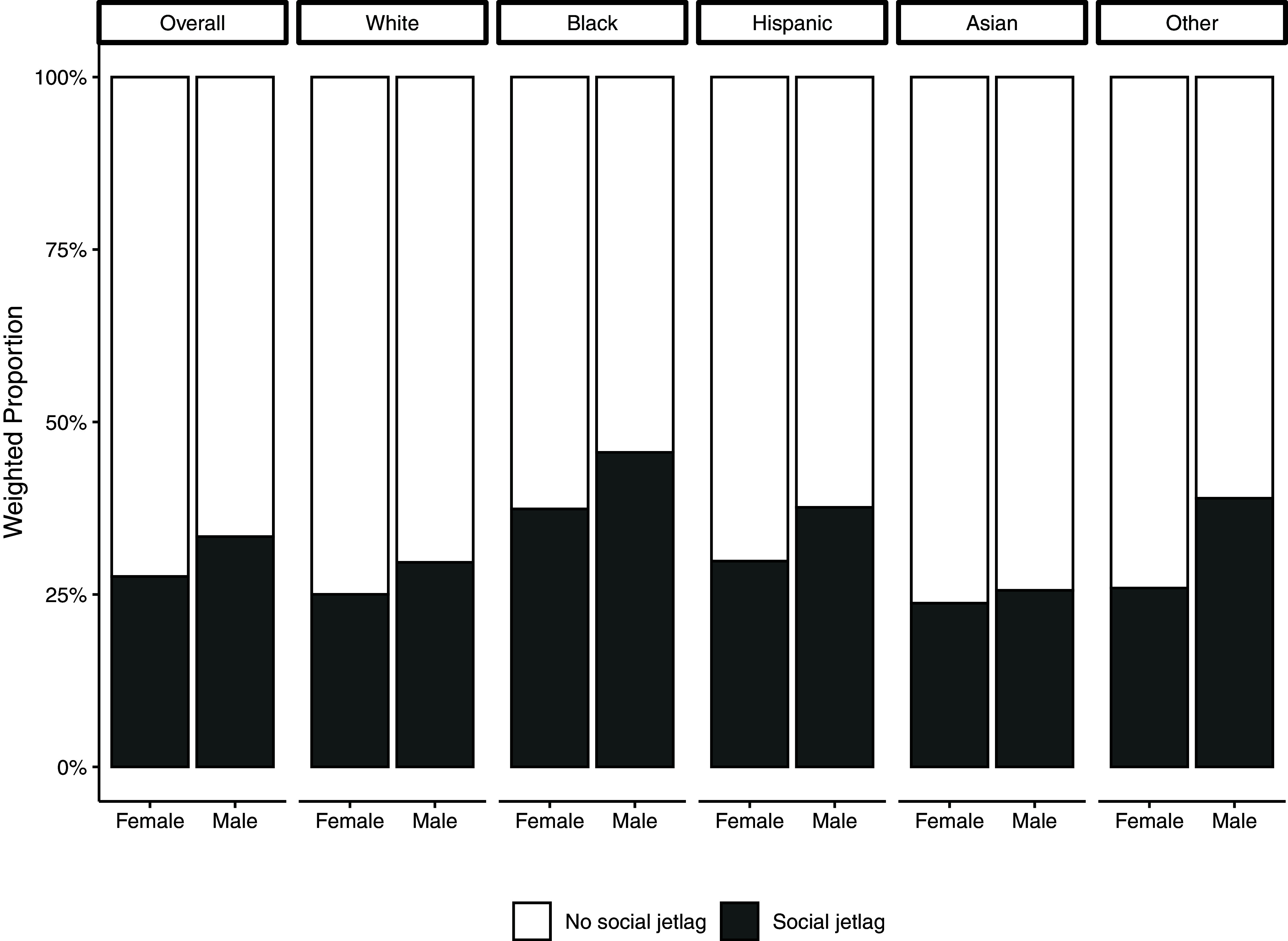
Title: Survey-weighted proportions of social jetlag participants by gender.

Demographic and health characteristics of survey participants are presented in Table 1. 31% of young adults in the overall sample had social jetlag. In addition, social jetlag was more prevalent among males than females (e.g. 34% males had social jetlag vs. 28% among females). Compared to those of other racial/ethnic groups, Black Americans were more likely to experience social jetlag, with more than 40% being affected (Fig. 2).

**Table 1.** Demographic and health characteristics of young adults (20–39yr) by social jetlag status

	Overall^a^ N = 1308 (%)	Social jetlag^a,^ ^b^ N = 421 (%)	No social jetlag^b^ N = 887 (%)
Sex			
Female	719 (51)	212 (28)	493 (72)
Male	637 (49)	209 (34)	394 (66)
Race/ethnicity			
White	437 (55)	114 (28)	323 (72)
Black	290 (13)	115 (41)	175 (59)
Hispanic	316 (20)	118 (33)	198 (67)
Asian	191 (7)	47 (25)	144 (75)
Other	74 (5)	27 (33)	47 (67)
Education			
More than high school	802 (65)	235 (30)	567 (70)
High school and below	506 (35)	186 (31)	320 (69)
Age (mean ± sd)	29.4 ± 5.7	29.8 ± 5.4	28.4 ± 5.8
Physical activity			
Any moderate/vigorous activity	1048 (84)	346 (32)	702 (68)
No moderate/vigorous activity	260 (16)	75 (23)	185 (77)
Tobacco use in the last 5 d			
Yes	347 (23)	134 (38)	213 (62)
No	961 (77)	287 (29)	674 (71)

a Only counts for categorical variables are unweighted. Proportions, means, and standard deviations are weighted according to the dietary two-day sample weight (WTDR2D).

b Social jetlag, a binary indicator, is defined as having at least 2-hr difference in weekday/workday and weekend sleep midpoints.

**Fig. 3.**
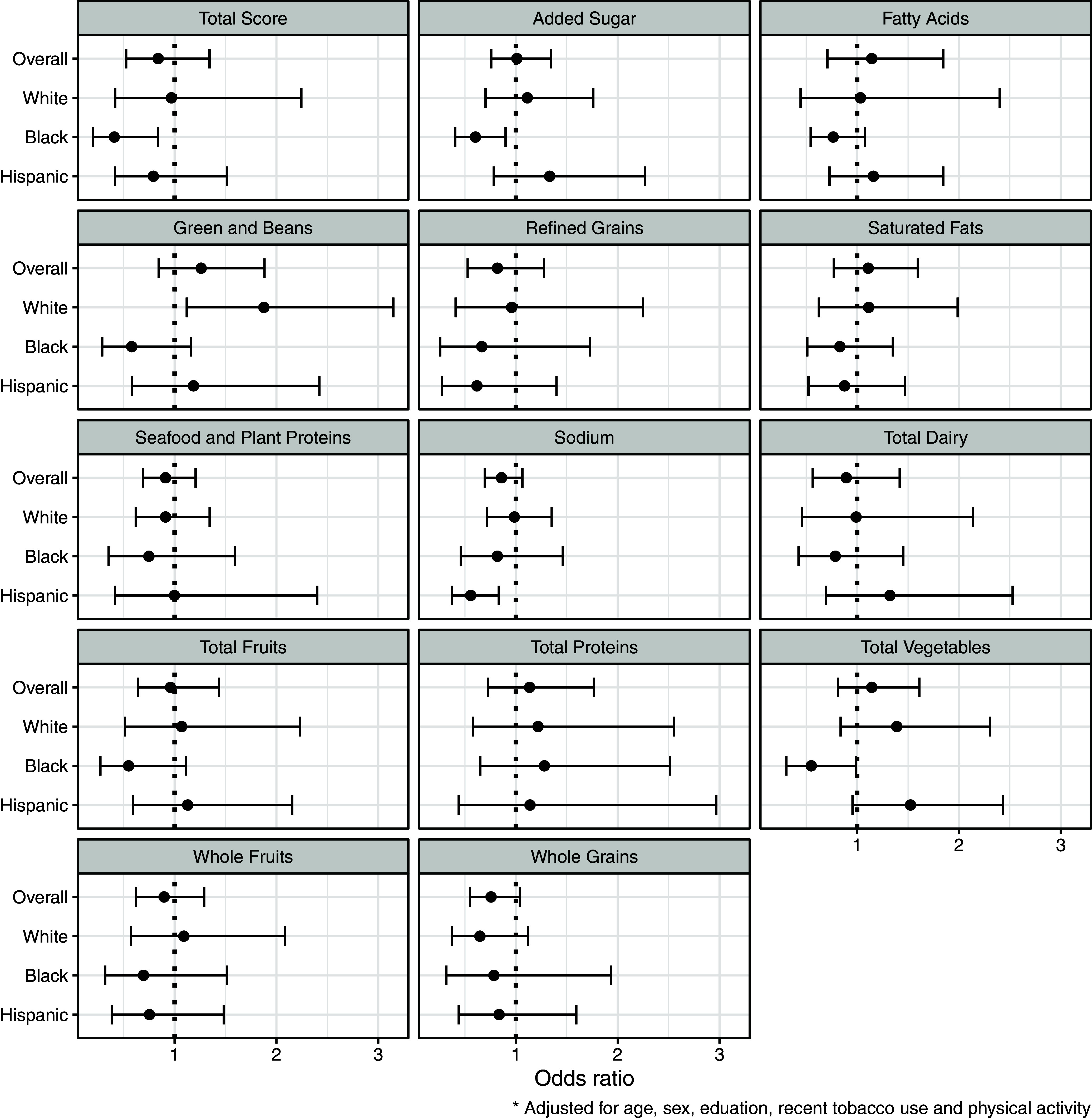
Title: Forest plots for overall HEI score and component scores, unstratified and stratified by race/ethnicity. ^1^Odds ratios are from adjusted ordinal logistic regression models with tertiles of diet scores as the categorical outcome and social jetlag as the exposure, adjusted for gender, race/ethnicity (only in the unstratified models), educational attainment, recent tobacco use and physical activity. Odds ratios<1 indicate lower diet quality (i.e. lower odds of being in the upper tertiles of diet quality).

For the overall sample, no significant relationships between social jetlag status and HEI-2015 scores were identified (Fig. 3).

However, there was effect modification of the relationship between social jetlag and diet quality according to race/ethnicity (Fig. 3). In particular, social jetlag was associated with lower overall diet quality scores for Black individuals (OR of being in higher-quality tertiles = 0.4 with 95% CI 0.2, 0.8). Regarding individual diet component scores, Black young adults with social jetlag had lower scores for Total Vegetables and Added Sugar (i.e. lower intake of vegetables and higher consumption of added sugar; OR = 0.6 with 95% CI 0.3, 1.0 and OR = 0.6 with 95% CI 0.4, 0.9, respectively). Among Hispanic young adults, social jetlag was associated with lower scores in the Sodium component (i.e. higher sodium intake; OR = –1.1, 95% CI –2.0, –0.2). Conversely, White young adults with social jetlag had higher scores in the Greens and Beans component (OR = 1.9, 95% CI 1.1, 3.2). There was no evidence for effect modification by gender (all P for interaction>0.05). In post hoc analysis when social jetlag was defined as ≥1 hour different between weekday and weekend sleep timing, social jetlag was associated with higher intake of sodium (OR = 0.7, 95% CI 0.5, 0.9).

## Discussion

Within this nationally representative sample of US young adults, social jetlag was not significantly associated with overall diet quality. However, there was effect modification by race/ethnicity. Specifically, Black Americans with social jetlag had lower overall diet quality scores and lower scores of the total vegetables and added sugar components (i.e. higher added sugar intake), while Hispanic Americans with social jetlag had lower scores on the sodium component (i.e. higher sodium intake). In contrast, White young adults with social jetlag had higher scores for the greens and beans component.

Overall, social jetlag was not an important determinant of diet quality, which contrasts with some young adult studies in Spanish,^(25)^ Japanese,^(26)^ and Brazilian populations.^(20)^ Nevertheless, a recent review^(33)^ of social jetlag and dietary intake that took into account findings of 17 studies provided a more cautious conclusion that social jetlag is *possibly* related to diet, given mixed findings especially for individual foods and nutrients. There could be a few reasons for the lack of an association in this study. First, diet quality is meant to capture total diet, and it could be that social jetlag primarily affects diet quality during the weekends. Furthermore, there could be compensatory mechanisms such that young adults consciously improve their diets and sleep during the week to ‘make up’ for poor diet and sleep on the weekends. Second, it is possible that we did not find strong associations between social jetlag and diet quality because there was insufficient variability in either diet quality or social jetlag in the young adult population as a whole.

Among racial/ethnic minorities, namely Black and Hispanic Americans, there was stronger evidence of associations between social jetlag and diet. Furthermore, these associations were in the expected directions, given that social jetlag has been previously linked with lower diet quality and higher consumption of individual foods/drinks such as sugar-sweetened beverages.^(20,25,26,34)^ Of note, social jetlag was overall higher in Black and Hispanic Americans than in other groups. There could be several reasons for this. Black and Hispanic Americans are more likely to live in neighbourhoods that may not be conducive to healthy sleep.^(35)^ Furthermore, racial/ethnic minorities suffer disproportionately from food insecurity^(36)^ and economic uncertainty,^(37)^ which could result in stress and changing circumstances (job, housing, etc.) that make it difficult or impossible to maintain consistent sleep schedules. In populations of US young adults not facing economic or job uncertainty, social jetlag could instead reflect different social or lifestyle decisions, e.g. choosing to stay up late on the weekends to socialize with friends. Thus, the reasons underlying social jetlag may differ between racial/ethnic subgroups of young adult populations in ways that modify relationships between sleep and diet. Indeed, we found that among White non-Hispanic young adults, social jetlag was unexpectedly associated with higher diet quality scores on the Greens and Beans component. While the reasons underlying this association are unclear, it is worth noting that at least one other study among US adolescents reported healthier behaviours among adolescents with higher social jetlag (although the study was conducted among Hispanic adolescents).^(38)^ In addition, our dataset showed that more physically active individuals were also more likely to have social jetlag which was not in line with expectation.^(39)^ Further research is needed to understand the drivers of social jetlag, especially within different subpopulations of young adults within the US.

There are both strengths and limitations to consider. This sample is large and reflective of the young adult population in the US, increasing the generalizability of findings. Second, the examination of effect modification by ethnic and racial minority groups uncovered potential disparities in sleep health and diet. Third, the comprehensive diet information allowed examination of specific dietary domains in relation to social jetlag. Some limitations include the self-report sleep timing that could be over- or under-estimated and the cross-sectional study design which prohibits the evaluation of the temporality of the associations. Furthermore, the two dietary recall days were not restricted to weekdays or weekends (i.e. it was not required that one recall day occur on a weekday and the other on a weekend), and the two-day survey weights were constructed to minimize differences in diet across the week. Given this design, we could not readily examine weekday-weekend differences.

In conclusion, within a nationally representative sample of US young adults, we found that social jetlag was related to certain indicators of lower diet quality among Black Americans and Hispanic Americans. These findings demonstrate that consistency of sleep timing from weekdays to weekends could constitute another barrier for consuming a healthy diet in the US, especially among Black and Hispanic Americans.
